# Towards a Noninvasive Intracranial Tumor Irradiation Using 3D Optical Imaging and Multimodal Data Registration

**DOI:** 10.1155/2007/62030

**Published:** 2007-04-15

**Authors:** R. Posada, Ch. Daul, D. Wolf, P. Aletti

**Affiliations:** ^1^Centre de Recherche en Automatique de Nancy (CRAN UMR 7039), Nancy-Université, CNRS, CAV, 2 avenue de la Forêt de Haye, 54516 Vandœuvre-Lès-Nancy, France; ^2^Instituto Tecnologico de Orizaba, avenida oriente 9 no. 852, Colonia Emiliano Zapata, 94320 Veracruz, Orizaba, Mexico

## Abstract

Conformal radiotherapy (CRT) results in high-precision tumor volume irradiation. In fractioned radiotherapy (FRT), lesions are irradiated in several sessions so that healthy neighbouring tissues are better preserved than when treatment is carried out in one fraction. In the case of intracranial tumors, classical methods of patient positioning in the irradiation machine coordinate system are invasive and only allow for CRT in one irradiation session. This contribution presents a noninvasive positioning method representing a first step towards the combination of CRT and FRT. The 3D data used for the positioning is point clouds spread over the patient's head (CT-data usually acquired during treatment) and points distributed over the patient's face which are acquired with a structured light sensor fixed in the therapy room. The geometrical transformation linking the coordinate systems of the diagnosis device (CT-modality) and the 3D sensor of the therapy room (visible light modality) is obtained by registering the surfaces represented by the two 3D point sets. The geometrical relationship between the coordinate systems of the 3D sensor and the irradiation machine is given by a calibration of the sensor position in the therapy room. The global transformation, computed with the two previous transformations, is sufficient to predict the tumor position in the irradiation machine coordinate system with only the corresponding position in the CT-coordinate system. Results obtained for a phantom show that the mean positioning error of tumors on the treatment machine isocentre is 0.4 mm. Tests performed with human data proved that the registration algorithm is accurate (0.1 mm mean distance between homologous points) and robust even for facial expression changes.

## 1. INTRODUCTION

### 1.1. Medical context

The goal of radiotherapy is to eradicate tumors while preserving the surrounding healthy organs as much as possible. Radiotherapy machines consist of X-ray sources turning around one axis and emitting ionizing beams destroying carcinogenic cells. One crucial task in radiotherapy is to know precisely the tumor position with respect to a 3D reference point called isocentre. During classical treatment, radiotherapists determine both the number and the distribution of the irradiation angles in order to control the energy distribution in the tumoral volume and to minimize the energy passing through the healthy regions. The more precise the patient placement is, the more efficient the radiotherapist's treatment protocols are.

**Figure 1:**
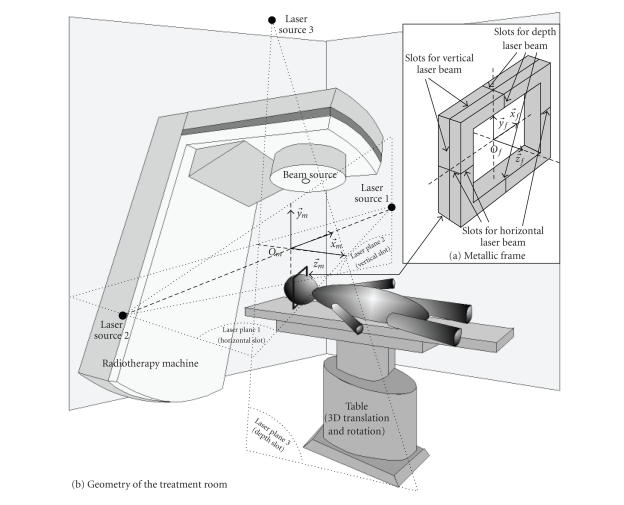
Principle of the frame-based method. (a) Geometry of the treatment room. (b) Frame geometry and coordinate system.

Treatment protocols depend on the organ to be irradiated. This paper focusses on intracranial tumor treatment. For such tumors, the positioning is usually based on metallic frames screwed on the patient's skull. The frame-based method is also employed by the radiotherapists of the oncology centre (Centre Alexis Vautrin, Nancy, France) associated to this work. The therapy always starts with a computer tomography (CT) or another similar examination, the frame being already screwed on the patient's head. The tumor borders, manually delineated in each image, are used to compute the 3D target volume and the lesion localization with regard to a coordinate system $\left(O_{f},{\overset{\to }{x}}_{f},{\overset{\to }{y}}_{f},{\overset{\to }{z}}_{f}\right)$ given by the frame. As shown in Figure 1, $\left(O_{f},{\vec{x}}_{f},{\vec{y}}_{f},{\vec{z}}_{f}\right)$ is defined by orthogonal slots machined into the frame. The ${\vec{x}}_{f}$, ${\vec{y}}_{f}$, and ${\vec{z}}_{f}$ vector axes take *O*_*f*_ (frame centre) as origin and pass through slot intersections. As both the frame and the tumor are visible in the CT, the lesion can be localized in $\left(O_{f},{\vec{x}}_{f},{\vec{y}}_{f},{\vec{z}}_{f}\right)$. As also illustrated in Figure 1, three laser beams sweep three orthogonal planes in the therapy room. The intersections of the three plane pairs support the orthogonal vectors of the irradiation machine coordinate system $\left(O_{m},{\vec{x}}_{m},{\vec{y}}_{m},{\vec{z}}_{m}\right)$. *O*_*m*_(isocentre) is the intersection point of the three planes. During the treatment, the patient's head is placed so that the laser planes fall into the slots. With this placement, $\left(O_{f},{\vec{x}}_{f},{\vec{y}}_{f},{\vec{z}}_{f}\right)$ and $\left(O_{m},{\vec{x}}_{m},{\vec{y}}_{m},{\vec{z}}_{m}\right)$ are superimposed. Knowing the tumor localization with respect to the frame, the table on which the patient lies is displaced to bring the lesion to the isocentre.

One obvious drawback of the frame-based method lies in the fact that the treatment is traumatic for the patient (the frame is screwed on the head). Moreover, the frame can neither be fixed for a long time on the patient's head nor screwed and unscrewed several times. Consequently, the irradiation must be performed in one unique session. Meanwhile, fractioned treatment (irradiation in several sessions) is more efficient than treatment performed in one fraction. Notably, the healthy organs are less damaged in fractioned radiotherapy (FRT) than in one session irradiations. The mean positioning errors of the best invasive frame methods are 1 mm [1]. With these small errors, conformal radiotherapy (CRT) can be efficiently used. CRT is a technique which results in very accurate target volume irradiation.

### 1.2. Previous work

In the case of intracranial tumors, only few solutions improving the patient's positioning step of radiotherapy treatment were proposed in the literature. Noninvasive frames were conceived and tested, the screws being for example replaced by bands surrounding the head and maintaining the frame [2]. Devices fixed in the ears and on the nose were also used to maintain the frame on the patient's head [3, 4]. These devices allow radiotherapists to use FRT since the frames can be fixed several times. Meanwhile, historical results [5] have shown that these Noninvasive frames lead to a rather inaccurate positioning, the daily set up variability ranging in [1–3] mm. These positioning errors are too high when radiotherapists want to take advantage of the high irradiation accuracy of CRT.

The positioning problem in radiotherapy is to find the geometrical relationship between the coordinate systems of the therapy machine and the diagnosis device (CT, etc.). This problem lies in the fact that the two devices are usually placed in different rooms of a hospital. One way to solve this problem is to place the diagnosis and treatment machines in the same room. For such solutions the geometrical relationship between the machines is known by construction and/or using calibration procedures. The known geometrical relationship is used either to displace the patient's table on rails [6] or with a robot [7]. These solutions lead to FRT and accurate positioning (1 mm error for [6]) but are usually far too expensive for most hospitals. For instance, a CT-scanner cannot always be dedicated to radiotherapy treatment only.

Another method employed for intracranial lesions [8] and prostate cancer [9, 10] is based on the use of portal images (PI) and digitally reconstructed radiographs (DRR) or simulated radiographs (SR). PI images are radiographs acquired during treatment. Since treatment involves high energy, the PI have poor contrast. DRR are artificial images computed with 3D CT data. The DRR are generated from the viewpoints of the PI. SR are radiographs acquired in simulation rooms having exactly the same geometry as treatment rooms, the irradiation sources being of low energy. The bone structures are the interesting information in the PI, DRR, and SR 2D planes. The disparity between the data of two modalities (IP and DRR [9, 10] or IP and SR [8]) is used to quantify the positioning quality. The bone structure segmentation and matching (registration) is done either visually or automatically. Such methods are not precise enough for CRT (1 cm error for [8] and 1.6 mm error for [9]). A noninvasive method was proposed by Meeks et al. [11] for intracranial tumors. The authors conceived a bite plate having on one of its extremities a molded part which is blocked by the patient's maxillary dentition. The bite plate supports aluminium spheres and infrared LEDs (ILEDs). Both the spheres and the tumors are visible in CT data. The tumor can be located in a coordinate system defined by the spheres. The ILEDs positions in the sphere coordinate system are obtained with a first calibration procedure. A 3D infrared sensor consisting of three cameras is fixed in the therapy room. The sensor position in the radiotherapy room is given by a second calibration. This sensor gives the ILEDs positions in the therapy room. Knowing the relative positions between the ILEDs and the spheres and the spheres and the tumors, it is possible to predict the tumor position in the treatment machine coordinate system. With this method, the mean positioning error is 1.11 mm. Among the Noninvasive solutions described in the literature, this method is one of the most accurate and can be used in CRT and FRT. Meanwhile, this accuracy was measured with respect to the results obtained for a classical frame-based method which was itself affected by errors. Moreover, the method is not suitable for people (small children and elderly people) who have missing teeth. A dedicated part (molded bite plate) must also be built for each patient.

Recently, Li et al. [12] proposed an interesting head positioning method based on 3D sensors fixed in the CT and therapy rooms. The algorithm principle can be divided into three parts consisting of a reference surface generation during CT-simulation, “controlled” patient face acquisitions in the therapy room, and data alignments providing the patient positioning parameters.

In the CT-room, the 3D sensor position is calibrated using a specially designed calibration plate. This calibration provides the geometrical link between the coordinate systems of the 3D sensor and of the CT-scanner (the 3D head surface and lesion positions are known with respect to a simulated isocentre and treatment machine coordinate system). During the CT-data acquisition, a 3D sensor is used to acquire points spread out over the patient's face. The corresponding 3D surface is placed in the planned (simulated) treatment position. The CT-face surface is not exactly the same as that given by the 3D sensor in the treatment room when face masks are used to immobilize the patient's head. Placing the 3D face surface acquired with 3D sensor in the simulation coordinate system (with the aim to replace the CT-surface) is one way to obtain a reference surface “comparable” to the face surface acquired in the treatment room. This placement is done with the calibration parameters.

Mandible or lip movements lead to nonnegligible changes in terms of facial expression. Li et al. project a light ray on the chin area and determine in real time skin/sensor distances. The mandible motions are small when the measured distances become stable (in such situations the authors verified that the acquired images were reliable). The treatment room sensor being calibrated with the same method as the CT-simulation sensor, the face point positions are known in the irradiation machine coordinate system.

The 3D surface obtained in the treatment room is then aligned with the reference surface using an iterative closest point algorithm. The geometrical parameters given by the alignment are used to adjust the head position.

Similar algorithms and sensors were used in [13] for breast lesion irradiation.

### 1.3. Objectives of the presented work

Considering the methods presented in the literature, the patient positioning algorithms proposed by Meeks et al. [11] and by Li et al. [12] are reference methods since they are Noninvasive and can be used in FRT. Meanwhile, the method of Meeks is not suitable for people (small children and elderly people) having missing teeth. The method of Li does not have this drawback. For this reason, a 3D sensor was chosen in the frame of our noninvasive patient positioning algorithm.

Facemasks are not always usable since some patients are allergic to masks or could not wear them because of a phobia. The positioning method must work with simple immobilization devices consisting of head supports and devices blocking the patient's forehead, ears, and/or mandibles. Thus, the smallest available reference face area for the positioning is the face region located between the bottom of the forehead and the bottom of the nose. With this constraint, cost and time related to the building of dedicated patient parts (face masks, dental supports, etc.) can be minimized.

Li et al. demonstrated that it is possible to position patients with submillimetre accuracy using 3D optical sensors. The aim of this contribution is to show that registration methods can lead to a robust patient positioning when using 3D sensors and simple immobilization devices. The method has to be precise even if the cutaneous face surface is not completely rigid (the surface shapes depend on facial expressions).

## 2. POSITIONING ALGORITHM

### 2.1. Algorithm principle

The difficulty relating to the patient positioning problem is due to the fact that the exact geometrical relationship between the CT coordinate system and that of the therapy machine is unknown. In other words, knowing only the tumor position in the CT coordinate system $\left(O_{\text{CT}},{\vec{x}}_{\text{CT}},{\vec{y}}_{\text{CT}},{\vec{z}}_{\text{CT}}\right)$ is not sufficient to determine the tumor position in the therapy machine coordinate system $\left(O_{m},{\vec{x}}_{m},{\vec{y}}_{m},{\vec{z}}_{m}\right)$.

In the case of the method used usually (invasive stereotactic frame), the relationship between $\left(O_{\text{CT}},{\vec{x}}_{\text{CT}},{\vec{y}}_{\text{CT}},{\vec{z}}_{\text{CT}}\right)$ and $\left(O_{m},{\vec{x}}_{m},{\vec{y}}_{m},{\vec{z}}_{m}\right)$ is known by using a third coordinate system related to the frame, namely, $\left(O_{f},{\vec{x}}_{f},{\vec{y}}_{f},{\vec{z}}_{f}\right)$. The positioning problem can be solved because the frame ensures two functions. First, the frame provides a coordinate system in which the tumor can be localized in the therapy room. Second, the frame is also able to localize the machine coordinate system. That is the reason why the frame must be exactly in the same position on the patient's head during the whole treatment.

For the proposed method, the frame is replaced by two devices, each device having one of the two functions of the frame. The first device is a 3D sensor which is fixed in the therapy room above the patient's table. This sensor acquires the 3D surface of the patient's face. This data is used to localize the tumor in the sensor coordinate system $\left(O_{3\text{DS}},{\vec{x}}_{3\text{DS}},{\vec{y}}_{3\text{DS}},{\vec{z}}_{3\text{DS}}\right)$. The second device is a calibration piece. The geometry of this piece allows us to determine the mathematical relationship between $\left(O_{3\text{DS}},{\vec{x}}_{3\text{DS}},{\vec{y}}_{3\text{DS}},{\vec{z}}_{3\text{DS}}\right)$ and $\left(O_{m},{\vec{x}}_{m},{\vec{y}}_{m},{\vec{z}}_{m}\right)$. The two devices are used in the frame of a two step algorithm.

**Step 1**

**Figure 2:**
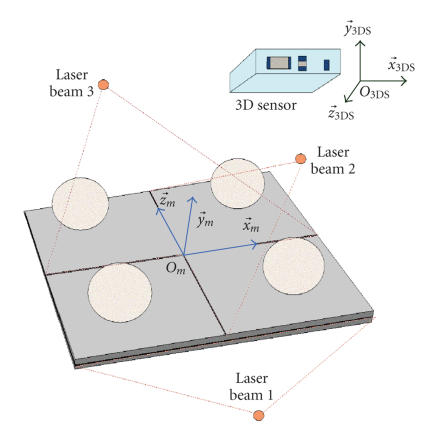
Calibration piece.

The calibration piece (see Figure 2) was specially designed for classical therapy rooms equipped with the laser system described in Figure 1. The calibration piece consists of four spheres fixed onto a plate in which orthogonal slots were machined. The plate is positioned on the patient's table so that the laser beams fall into the slots. In this situation, the exact positions of the four sphere centres are known by construction in $\left(O_{m},{\vec{x}}_{m},{\vec{y}}_{m},{\vec{z}}_{m}\right)$. An image of the calibration piece is acquired with the 3D sensor and the sphere centre coordinates are computed in $\left(O_{3\text{DS}},{\vec{x}}_{3\text{DS}},{\vec{y}}_{3\text{DS}},{\vec{z}}_{3\text{DS}}\right)$. It is possible to find analytically the ${\tilde{T}}_{3\text{DS},m}$ transformation linking $\left(O_{3\text{DS}},{\vec{x}}_{3\text{DS}},{\vec{y}}_{3\text{DS}},{\vec{z}}_{3\text{DS}}\right)$ to $\left(O_{m},{\vec{x}}_{m},{\vec{y}}_{m},{\vec{z}}_{m}\right)$ if, for a given calibration piece position, the sphere centre coordinates are known in the coordinate systems of both the 3D sensor and the therapy machine.

**Step 2**

During standard intracranial cancer treatment, the head borders are marked in the CT images so that the 3D head surface is systematically available. The registration of the 3D head data with the 3D face data acquired in the therapy room gives the ${\tilde{T}}_{\text{CT},3\text{DS}}$ geometrical transformation linking $\left(O_{\text{CT}},{\vec{x}}_{\text{CT}},{\vec{y}}_{\text{CT}},{\vec{z}}_{\text{CT}}\right)$ to $\left(O_{3\text{DS}},{\vec{x}}_{3\text{DS}},{\vec{y}}_{3\text{DS}},{\vec{z}}_{3\text{DS}}\right)$.

The ${\tilde{T}}_{3\text{DS},m}$ and ${\tilde{T}}_{\text{CT},3\text{DS}}$ transformations being matrices, the global transformation matrix ${\tilde{T}}_{\text{CT},m}={\tilde{T}}_{3\text{DS},m}\times {\tilde{T}}_{\text{CT},3\text{DS}}$ is sufficient to compute a given point position in $\left(O_{m},{\vec{x}}_{m},{\vec{y}}_{m},{\vec{z}}_{m}\right)$ with only its corresponding position known in $\left(O_{\text{CT}},{\vec{x}}_{\text{CT}},{\vec{y}}_{\text{CT}},{\vec{z}}_{\text{CT}}\right)$. Since both the 3D sensor and the CT-scanner provide data without spatial distortion and with the same isotropic scale factor of 1, ${\tilde{T}}_{\text{CT},3\text{DS}}$ and ${\tilde{T}}_{3\text{DS},m}$ are isometries (matrices containing only 3D translations and 3D rotations).

### 2.2. Data and 3D sensor description

In the CT-modality, data sets are typically represented by about 2000 points spread out over the whole cutaneous surface of the patient's head. The voxel size of the CT-scanner equals 0.313 mm × 0.313 mm × 2 mm.

The measurement principle of the 3D sensor^1^ . fixed in the therapy room is based on the structured light (visible light modality). The sensor is able to acquire data without any strong and particular constraints (no change in the lighting conditions, etc.). The face/3D sensor distance must only be approximatively 1 m. The typical data provided by the sensor is clouds of about 7000 points distributed over the patient's face. The field of view equals 210 mm × 320 mm for a depth of view of 100 mm. The sensor has a spatial resolution of 2 mm, 1 mm, and 0.2 mm for the ${\vec{x}}_{3\text{DS}}$, ${\vec{y}}_{3\text{DS}}$, and ${\vec{z}}_{3\text{DS}}$ axes, respectively.

### 2.3. 3D sensor calibration

The calibration starts with an acquisition of the calibration piece placed in such a way on the patient's table that the laser beams fall into the slots. In the first calibration step, the sphere centre coordinates are determined in $\left(O_{3\text{DS}},{\vec{x}}_{3\text{DS}},{\vec{y}}_{3\text{DS}},{\vec{z}}_{3\text{DS}}\right)$. The second step consists in the search for the analytical relationship $\left({\tilde{T}}_{3\text{DS},m}\right)$ between the sphere centre positions in the sensor coordinate system and the same positions in $\left(O_{m},{\vec{x}}_{m},{\vec{y}}_{m},{\vec{z}}_{m}\right)$.

#### 2.3.1. Sphere centre computation

For each 3D point, the sensor gives both position information and a color value. To take advantage of the color data, the calibration piece is put on black fabric. It is noticeable in Figure 2 that the color of the spheres is bright, while the plate is dark. With the color intensity information, it is easy to separate the sphere points from the other points (image background and plate points).

**Figure 3:**
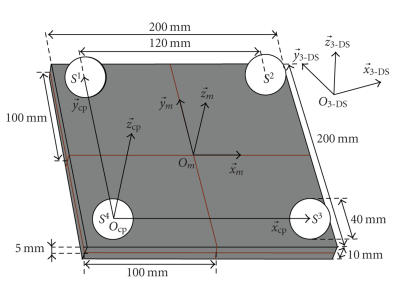
Calibration piece dimensions and coordinate systems.

The geometry of the calibration piece is well known: 40 mm sphere diameters and 120 mm distances between neighbouring spheres (see Figure 3). These values, and all others relating to the calibration piece geometry, are known by construction with a 0.01 mm accuracy. During the calibration, the 3D points are sorted in four groups each corresponding to one sphere. The sorting is performed as follows: if the distance between the point currently treated and a point of a group is smaller or equal to 40 mm, then the current point is assigned to the tested point group.

 (the 3D sensor reconstructs the points with small errors), the points are not exactly located on a sphere. *S* ^*n*^ is the *n*th sphere (*n* = 1,2,3,4) of radius*r* and has a centre *C* ^*n*^ with coordinates (*X* ^*n*^_3DS_, *Y* ^*n*^_3DS_, *Z* ^*n*^_3DS_) in $\left(O_{3\text{DS}},{\vec{x}}_{3\text{DS}},{\vec{y}}_{3\text{DS}},{\vec{z}}_{3\text{DS}}\right)$. If the *i*th point *p* ^*i,n*^ (*i* ∈ [1*I*_n_], *I*_n_ point number of group*n* ), of coordinates (*X* ^*i,n*^_3DS_, *Y* ^*i,n*^_3DS_, *Z* ^*i,n*^_3DS_), belongs to the sphere*S* ^*n*^ , then (1) is verified:

(1) $${\left(x_{\text{3DS}}^{i,n}-x_{\text{3DS}}^{n}\right)}^{2}+{\left(y_{3-D}^{i,n}-y_{\text{3DS}}^{n}\right)}^{2}+{\left(z_{\text{3DS}}^{i,n}-z_{\text{3DS}}^{n}\right)}^{2}=r^{2}.$$

The coordinates of centre *C* ^*n*^ are determined by minimizing the functional *ε*_*n*_ given in (2),

(2) $$\varepsilon_{n}=\sum_{i=1}^{I_{n}}\left|{\left(x_{3\text{DS}}^{i,n}-x_{3\text{DS}}^{n}\right)}^{2}+{\left(y_{3-D}^{i,n}-y_{3\text{DS}}^{n}\right)}^{2}+\left(z_{3\text{DS}}^{i,n}-z_{3\text{DS}}^{n}\right)-r^{2}\right|.$$

The initial value of the centre coordinates are given by the gravity centre of all the points of a group. The simplex [14] is used as optimization method since this algorithm is accurate and converges quickly towards the minimum when the solutions are close to the initial values.

#### 2.3.2. Calibration matrix determination

The ${\tilde{T}}_{3\text{DS},m}$ homogeneous matrix, which provides the coordinates (*X*_*m*_ *Y*_*m*_ *Z*_*m*_) of a point in the therapy machine coordinate system using the coordinates (*X*_3DS_ *y*_3DS_ *Z*_3DS_ ) of the same point in 3D sensor coordinate system, consists of nine *r* ^cal^_*i*_ rotation parameters and three *t* ^cal^_*j*_ translation parameters (see (3)):

(3) $$\left(\begin{array}{c} x_{m}\\ y_{m}\\ z_{m}\\ 1 \end{array}\right)=\underset{{\tilde{T}}_{3\text{DS},m}}{\underset{︸}{\left(\begin{array}{cccc} r_{1}^{cal} & r_{2}^{cal} & r_{3}^{cal} & t_{x}^{cal}\\ r_{4}^{cal} & r_{5}^{cal} & r_{6}^{cal} & t_{y}^{cal}\\ r_{7}^{cal} & r_{8}^{cal} & r_{9}^{cal} & t_{z}^{cal}\\ 0 & 0 & 0 & 1 \end{array}\right)}}\left(\begin{array}{c} x_{3\text{DS}}\\ y_{3\text{DS}}\\ z_{3\text{DS}}\\ 1 \end{array}\right).$$

As shown in Figure 3, the three spheres *S* ^1^, *S* ^3^, and *S* ^4^ define the calibration piece coordinate system $\left(O_{\text{cp}},{\vec{x}}_{\text{cp}},{\vec{y}}_{\text{cp}},{\vec{z}}_{\text{cp}}\right)$. The fourth sphere *S* ^2^ is only used to check the calibration results consistency. The rotation parameters *r* ^*i*^_cal_ express the point with coordinates *X*_3DS_ *Y*_3DS_ *Z*_3DS_ in a rotated coordinate system having the same origin as the 3D sensor coordinate system but with axes parallel to those of $\left(O_{\text{cp}},{\vec{x}}_{\text{cp}},{\vec{y}}_{\text{cp}},{\vec{z}}_{\text{cp}}\right)$. The *r* ^cal^_*i*_ parameter values are given by (4) and depend on the sphere centre coordinates of (1). *d*_1_, *d*_2_ and *d*_1_, *d*_2_ are the norms of ${\vec{x}}_{\text{cp}}$, ${\vec{y}}_{\text{cp}}$, and ${\vec{z}}_{\text{cp}}$, respectively,

(4) $$\begin{array}{ll} r_{1}^{cal} & =\frac{x_{3\text{DS}}^{3}-x_{3\text{DS}}^{4}}{d_{1}},r_{2}^{cal}=\frac{y_{3\text{DS}}^{3}-y_{3\text{DS}}^{4}}{d_{1}},\\ r_{3}^{cal} & =\frac{z_{3\text{DS}}^{3}-z_{3\text{DS}}^{4}}{d_{1}},r_{4}^{cal}=\frac{x_{3\text{DS}}^{1}-x_{3\text{DS}}^{4}}{d_{2}},\\ r_{5}^{cal} & =\frac{y_{3\text{DS}}^{1}-y_{3\text{DS}}^{4}}{d_{2}},r_{6}^{cal}=\frac{z_{3\text{DS}}^{1}-z_{3\text{DS}}^{4}}{d_{2}},\\ r_{7}^{cal} & =\frac{\left(y_{3\text{DS}}^{3}-y_{3\text{DS}}^{4})(z_{3\text{DS}}^{1}-z_{3\text{DS}}^{4}\right)}{d_{1}d_{2}}\\ -\frac{\left(y_{3\text{DS}}^{1}-y_{3\text{DS}}^{4})(z_{3\text{DS}}^{3}-z_{3\text{DS}}^{4}\right)}{d_{1}d_{2}},\\ r_{8}^{cal} & =\frac{\left(x_{3\text{DS}}^{1}-x_{3\text{DS}}^{4})(z_{3\text{DS}}^{3}-z_{3\text{DS}}^{4}\right)}{d_{1}d_{2}}\\ -\frac{\left(x_{3\text{DS}}^{3}-x_{3\text{DS}}^{4})(z_{3\text{DS}}^{1}-z_{3\text{DS}}^{4}\right)}{d_{1}d_{2}},\\ r_{9}^{cal} & =\frac{\left(x_{3\text{DS}}^{3}-x_{3\text{DS}}^{4})(y_{3\text{DS}}^{1}-y_{3\text{DS}}^{4}\right)}{d_{1}d_{2}}\\ -\frac{\left(x_{3\text{DS}}^{1}-x_{3\text{DS}}^{4})(z_{3\text{DS}}^{3}-z_{3\text{DS}}^{4}\right)}{d_{1}d_{2}},\\ d_{1}=[ & {\left(x_{3\text{DS}}^{3}-x_{3\text{DS}}^{4}\right)}^{2}+{\left(y_{3\text{DS}}^{3}-y_{3\text{DS}}^{4}\right)}^{2}\\ +{\left(z_{3\text{DS}}^{3}-z_{3\text{DS}}^{4}\right)}^{2}{]}^{1/2},\\ d_{2}=[ & {\left(x_{3\text{DS}}^{1}-x_{3\text{DS}}^{4}\right)}^{2}+{\left(y_{3\text{DS}}^{1}-y_{3\text{DS}}^{4}\right)}^{2}\\ +{\left(z_{3\text{DS}}^{1}-z_{3\text{DS}}^{4}\right)}^{2}{]}^{1/2}. \end{array}$$

As formulated in (5), two 3D translations define the global translation linking the 3D sensor and the therapy machine coordinate systems. The parameters of translation 1 are directly related to the coordinates (*X* ^4^_3DS_,*Y* ^4^_3DS_, *Z* ^4^_3DS_) of the *S* ^4^ sphere centre position while translation 2 is completely defined by the calibration piece dimensions. Translation 2 gives the distances between the origins of the calibration piece and the therapy machine coordinate systems along the *x*- and *y*- and *z*-axes:

(5) $$\left(\begin{array}{c} t_{x}^{cal}\\ t_{y}^{cal}\\ t_{z}^{cal} \end{array}\right)=\underset{\text{translation 1}}{\underset{︸}{\left(\begin{array}{c} -x_{3\text{DS}}^{4}\\ -y_{3\text{DS}}^{4}\\ -z_{3\text{DS}}^{4} \end{array}\right)}}+\underset{\text{translation 2}}{\underset{︸}{\left(\begin{array}{c} -60\\ -60\\ 25 \end{array}\right)}}.$$

### 2.4. 3D data registration

An analysis of review papers dealing with medical image registration [15–17] shows that the superimposition of 3D CT data and 3D structured light data is an application that is hardly ever studied.

#### 2.4.1. General considerations

Let us consider *I*_*m*_(*X*_*m*_, *Y*_*m*_, *Z*_*m*_) and *I*_*t*_(*X*_*t*_, *Y*_*t*_, *Z*_*t*_) as two 3D images containing homologous structures *D*_*m*_ and *D*_*t*_ extracted from the images with the segmentation algorithms *f*_*m*_ and *f*_*t*_. The *D*_*t*_ data is transformed with the aim of superimposing it with the *D*_*m*_model data. In other words, the registration procedure consists in finding the parameters *θ* of the ${\tilde{T}}_{\text{CT},3\text{DS}}$ transformation such as $D_{m}={\tilde{T}}_{\text{CT},3\text{DS}}(D_{t})$. The homologous structures are superimposed with an optimization method Ψ which minimizes a similarity measure *S*. The principle of the registration method providing the optimal ${\tilde{T}}_{\text{CT},3\text{DS}}$ transformation is mathematically formulated in (6):

(6) $${\tilde{T}}_{\text{CT},3\text{DS}}=\arg\underset{\theta \in \Theta |\Psi }{\min}S(T(\underset{D_{t}}{\underset{︸}{f_{t}(I_{t})}}),\underset{D_{m}}{\underset{︸}{f_{m}(I_{m})}}).$$

In our patient positioning application, *D*_*m*_ and *D*_*t*_ are point clouds directly provided by the sensors of the two modalities. No *f*_*m*_ and *f*_*t*_ segmentation algorithms are needed to extract the homologous structures. The advantage of our method is that the errors inherent in the segmentation algorithms are avoided.

It is noticeable that both the 3D point densities (see Section 2.2 ) and the 3D surface sizes are different for the two modalities. The model surface (*D*_*m*_ data set, patient's face of the visible light modality) is completely a part of the transformed surface (*D*_*t*_ data set, patient's head of the CT-modality) when the two data sets are registered.

The registration requires the definition of four mathematical entities, namely, the transformation type, the similarity measure *S*, the transformation space Θ giving the limits of the *θ*-parameters, and the search strategy (optimization Ψ).

#### 2.4.2. Transformation type

As justified in Section 2.1, the transformation parameters are those of an isometry. The choice of the transformation type of ${\tilde{T}}_{\text{CT},3\text{DS}}$ was also realized on the assumption that a patient can make “similar enough facial expressions” during the CT-scan and the data acquisition with the 3D sensor (the impact of facial expression differences on the registration is discussed in Section 3.5).

The homogenous matrix ${\tilde{T}}_{\text{CT},3\text{DS}}$, used to determine the coordinates (*X*_3DS_, *Y*_3DS_, *z*_3DS_) of a point (tumor) in $\left(O_{3\text{DS}},{\vec{x}}_{3\text{DS}},{\vec{y}}_{3\text{DS}},{\vec{z}}_{3\text{DS}}\right)$ using the coordinates (*X*_3 CT_, *Y*_3 CT_, *Z*_3 CT_) of the same point in $\left(O_{3\text{CT}},{\vec{x}}_{3\text{CT}},{\vec{y}}_{3\text{CT}},{\vec{z}}_{3\text{CT}}\right)$, consists of the *t* ^reg^_*x*_, *t* ^reg^_*y*_, and *t* ^reg^_*z*_ translation parameters and of nine , *r* ^reg^_*i*_ rotation parameters. The rotation parameters are defined with the Euler angles (for the Euler angles, the so-called “ *x*-convention” is used: the first rotation is by an angle *ψ* about the ${\vec{z}}_{\text{CT}}$-vector, the second is by an angle *θ* ∈ [0,*π*] about the new ${\vec{x}}_{\text{CT}}$-vector, and the third is by an angle *ϕ* about the new ${\vec{z}}_{\text{CT}}$-vector).

#### 2.4.3. Similarity measurement

During the registration of two surfaces, the similarity (superimposition degree) can be assessed by measuring a distance between the surfaces. In the case of surfaces represented by point clouds, the bottleneck distance [18], the Hausdorff distance (Hd) [19], the directed Hausdorff distance (dHd), or the combination of several of these distances [20] are often suitable. For a given application, a distance measure can be chosen according to the following criteria.

**Data set type**

A given measure is suitable or not depending on whether the two surfaces are represented by a similar or a different point number. The fact that the surfaces to be matched have the same size or not is another decision criterion.

**Robustness against perturbations**

Surfaces partially hidden, noise affecting the positions of all the points, or data sets with outliers, influence more or less the similarity measure correctness depending on the chosen measure.

**Required transformation invariance**

The measure has to exhibit appropriate properties according to the type of the geometrical transformation used in the registration scheme. For example, for isometries or affine transformations, *d*(*A,B*) = *d*(*T*(*A*),*T*(*B*)) must be verified, *d* being the distance between two data sets *A* and *B*.

The bottleneck distance is suitable for data sets consisting of the same number of points. *D*_*m*_ and *D*_*t*_ being of different sizes, the bottleneck distance cannot be used in our application. Both the Hd and the dHd are suitable for data sets consisting in different point numbers. They are also invariant under isometries, and are robust against noise affecting the point positions. The dHd (*h*(*A, B*) defined in (7)) is the greatest Euclidean distance chosen between all the smallest Euclidean distances from a point *a* of the data set *A* to all points *b* of the data set *B*. The Hd (*H*(*A, B* of (8)) is computed using the dHd,

(7) $$h(A,B)=\underset{a\in A}{\max}\underset{b\in B}{\;\min}∥a-b∥,$$

(8) H(A,B)=max⁢ (h(A,B),  h(B, A)).

One advantage of the dHd, with respect to the Hd, lies in the fact that the *h*(*A, B*) distance is more robust against occlusions than the *H*(*A,B*) distance. In our positioning problem, the *D_m_* model data set (patient's face) represents a smaller 3D surface than the *D_t_* data set (patient's head). Indeed, the back of the patient's head is hidden for the 3D sensor fixed in the therapy room while the whole head is acquired in the CT-modality. Robustness against occlusions was the first criterion for choosing the dHd.

The second advantage of the dHd lies in the properties of the *h*(*A, B*) and *H*(*A, B*) distances. It is well known that the *H*(*A, B*) distance is a metric. This means in particular that *H*(*A,A*) = 0 (identity) and that *H*(*A, B*) + *H*(*A,C*) ≥ *H* (*B*, *C*)(strong triangle inequality) are verified by the Hd. Symmetry (*H*(*A, B*) = *H*(*B, A*)) follows from the identity and strong triangle inequality. Symmetry is a propriety which is required in many matching problems. The strong triangle inequality is not verified by the dHd and consequently *h*(*A, B*) ≠ *h*(*B, A*). For the proposed application, if the two data sets are best registered then *D_m_* is included in *D_t_*. This means that for registered data, *h*(*D_t_*, *D_m_*) is greater than zero and *h*(*D_m_*, *D_t_*) equals 0 (in fact due to coordinate discretization, this latter value is small but never null). The dHd has also been chosen because it is interesting to have a similarity measure *h*(*D_m_*, *D_t_*) whose value is very small when the data is registered and which becomes monotonically greater when the surfaces move apart (in our application, the increasing of the similarity measure is not monotonic for the Hd).

#### 2.4.4. Feature space limits

**Figure 4:**
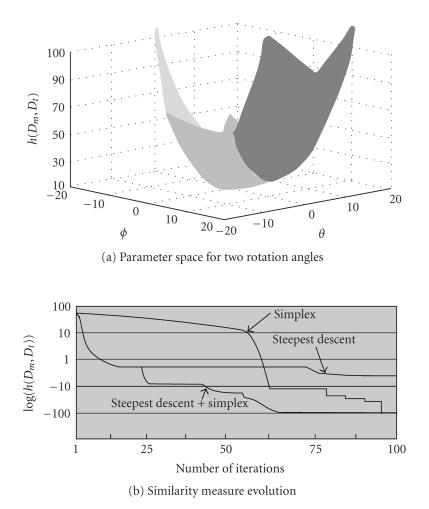
Appropriateness of the dHd. (a) Feature space for two rotation angles given in degrees. The *D*_*m*_ (3D sensor data) and *D*_*t*_ (CT modality) point sets were acquired for a phantom (plaster head). The *h*(*D*_*m*_, *D*_*t*_) surface is not only convex for these two angles, but also for all other parameters of the isometry. (b) Similarity measure evolution. The decimal logarithm values of *h*(*D*_*m*_, *D*_*t*_) are given for each iteration of the optimization. The combination of the steepest gradient and the simplex allows both a fast and accurate convergence.

The interesting feature limits are those defining a parameter space Θ having a unique minimum and a convex similarity measure surface (see Figure 4(a)). The dHd measure is very robust against translations. Theoretically, there are no translation limits beyond which the surface convexity is affected. For the two *D_m_* and *D_t_* data modalities, it has also been verified experimentally that *h*(*D_m_*, *D_t_*) decreases monotonically for rotation angles ranging between [−20° , 20°]. The patient's positions and the angles of view being approximately the same in the CT-scanner and on the radiotherapy table, only small rotation angles have to be considered for the registration. In this situation, the six-dimensional parameter space consisting of three translations and three rotations is effectively convex.

#### 2.4.5. Minimization method

Experiments proved that the data of the two modalities lead to a quasiconvex hypersurface (instead of an ideal convex surface) having one global minimum in the six-dimensional parameter space. Indeed, small local minima affect the hypersurface. A steepest descent algorithm is first used since this method converges quickly towards the solution whereas small local minima are avoided. As this algorithm only comes near to the global minimum (without reaching it), the simplex algorithm has been then used for obtaining the final ${\tilde{T}}_{\text{CT},3\text{DS}}$. The simplex algorithm is robust and accurate if the initialization is close to the solution (see Figure 4(b)).

#### 2.4.6. Inherent accuracy of the registration algorithm

**Figure 5:**
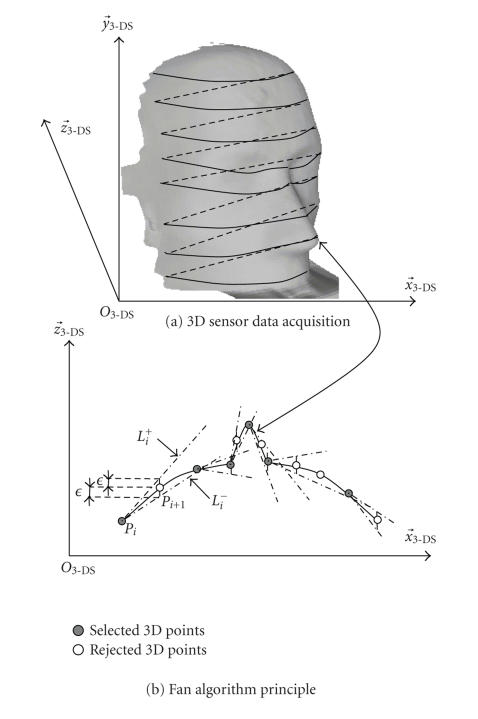
Data down-sampling algorithm.

Data was acquired for a phantom (plaster head, see Figure 5(a) ) with the 3D sensor in order to assess the inherent accuracy of the registration algorithm. A known ${\tilde{T}}_{\text{test}}$ transformation was applied to this data set *D*_*m*_ , taken as model, to obtain the transformed data *D*_*t*_. The registration algorithm was then used to superimpose *D*_*t*_ on *D*_*m*_. For the ${\tilde{T}}_{\text{CT},3\text{DS}}$ matrix obtained in this way, one should ideally have ${\tilde{T}}_{\text{CT},3\text{DS}}$ = ${\tilde{T}}_{\text{test}}^{-1}$.

The parameter values of the ${\tilde{T}}_{\text{test}}^{-1}$ transformation are given in the first column of Table 1. The second column of Table 1 gives the value differences between the parameters of ${\tilde{T}}_{\text{CT},3\text{DS}}$ and the corresponding ones of ${\tilde{T}}_{\text{test}}^{-1}$. For the second column, the ${\tilde{T}}_{\text{CT},3\text{DS}}$ transformation was computed with the whole points of the *D*_*m*_ and *D*_*t*_ data sets (without point down-sampling, see Section 2.4.7). The greatest differences were about 1° and several hundredth of mm for, respectively, the three rotation angles (*ψ*, *θ* and *ϕ*) and the translations (*t* ^reg^_*x*_, *t* ^reg^_*y*_ and *t* ^reg^_*z*_). These differences lead to a mean registration error of 0.03 mm (mean Euclidian distance between homologous points of *D*_*m*_ and *D*_*t*_ transformed by ${\tilde{T}}_{\text{CT},3\text{DS}}$, namely, ${\tilde{T}}_{\text{CT},3\text{DS}}({\tilde{T}}_{\text{test}}(D_{m}))$). This test, performed with monomodal data, prove that the registration algorithm has high inherent accuracy.

#### 2.4.7. Data down-sampling

The results obtained for the registration algorithm are satisfactory in terms of inherent accuracy but are not acceptable in the clinical case since the computation of ${\tilde{T}}_{\text{CT},3\text{DS}}$ requires about 4 hours on a PC with a 3.2 GHz Pentium IV processor with 2 gigabytes of RAM (the programs were written in C). This time is high since the application of the dHd to the two data sets consisting, respectively, of about 7000 3D points (visible light modality, *D*_*m*_) and 2000 points (CT-modality, *D*_*t*_) implies the computation of 14 million Euclidian distances. One solution to reduce the registration time is to diminish the point number of one modality. The visible light modality has been chosen since the *D*_*m*_ data set is the one with the most of the points.

The structured light-based sensor stores the 3D points camera line by camera line, each line having a constant *y*_3DS_ value (see Figure 5(a)). A down-sampling algorithm (Fan algorithm [21]) whose principle is sketched in Figure 5(b) is used to eliminate points characterized by a low curvature. Two consecutive points (*P*_*i*_ and *P*_*i*+1_) and a height value *ε* define two lines *L* ^+^_*i*_ and *L* ^−^_*i*_ with a given aperture angle depending on *ε*. The selected points are *P*_*i*_ and the last point lying between the two lines. The last point becomes the new *P*_*i*_ and the algorithm is repeated until the last point on the line is reached.

By giving at *ε* the values of 0.01 (2014 remaining points for *D*_*m*_ ), 0.1 (808 points), and 0.5 (406 points), the computation time of 4 hours (*ε* = 0, whole data set *D*_*m*_ of 7060 points) falls, respectively, to 50 minutes, 12 minutes, and 2 minutes. The last time, also obtained with a Pentium IV processor, is acceptable in the frame of standard treatment protocols. Moreover, faster computers can be used if this time must still be reduced. It is noticeable in Table 1 that the data down-sampling with the *ε* values reported here had a very weak influence on the parameters of ${\tilde{T}}_{\text{CT},3\text{DS}}$ and on the inherent registration algorithm accuracy.

### 2.5. Tumor position in the therapy room

Finally, the(*X* ^tum^_*m*_, *Y* ^tum^_*m*_, *z* ^tum^_*m*_) tumor position in the therapy machine coordinate system can easily be computed with the (*X* ^tum^_CT_, *Y* ^tum^_CT_, *Z* ^tum^_CT_) tumor position in the CT coordinate system and the global transformation matrix ${\tilde{T}}_{\text{CT},3\text{DS}}$ (see (9)):

(9) $$\left(\begin{array}{c} x_{m}^{\text{tum}}\\ y_{m}^{\text{tum}}\\ z_{m}^{\text{tum}}\\ 1 \end{array}\right)=\underset{{\tilde{T}}_{\text{CT},3\text{DS}}}{\underset{︸}{{\tilde{T}}_{3\text{DS},m}{\tilde{T}}_{\text{CT},3\text{DS}}}}\left(\begin{array}{c} x_{\text{CT}}^{\text{tum}}\\ y_{\text{CT}}^{\text{tum}}\\ z_{\text{CT}}^{\text{tum}}\\ 1 \end{array}\right).$$

## 3. EXPERIMENTS AND RESULTS

### 3.1. Simulation room description

During standard treatment, the patient positioning is first realized in a simulation room in order to assess the positioning accuracy and to check the dose distribution. The simulation room is geometrically identical to the treatment room. The two rooms are also equipped with the same devices. In particular, the simulation machine isocentre is also visualized by three laser beams.

**Figure 6:**
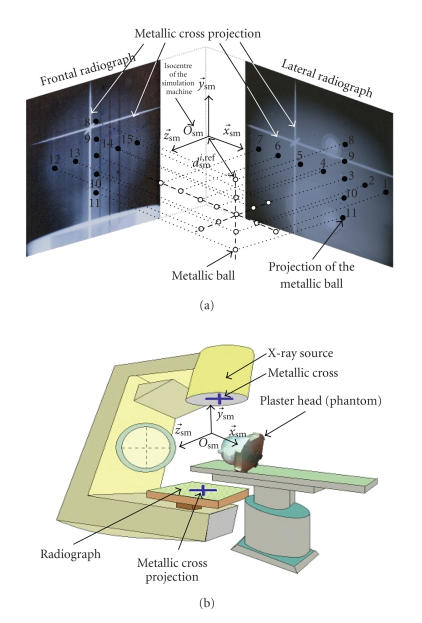
Simulation room. (a) Orthogonal control radiographs. (b) Room geometry.

However, between the two rooms there is a major difference related to the energy emitted by the irradiation sources. The linear accelerator of the treatment room is characterized by high energy whereas the source of the simulation machine is suitable to the realization of radiographic films (control radiographs). Such radiographs are generally taken for two well-defined viewpoints (see Figure 6).

As for the therapy machine coordinate system, the $\left(O_{\text{sm}},{\vec{x}}_{\text{sm}},{\vec{y}}_{\text{sm}},{\vec{z}}_{\text{sm}}\right)$ simulation machine coordinate system is completely defined and visualized by the laser beams. The two control radiographs are orthogonal since the first radiograph is parallel to the plane defined by the $\left({\vec{x}}_{\text{sm}},{\vec{y}}_{\text{sm}}\right)$ axis pair, while the second radiograph is parallel to the (${\vec{z}}_{\text{sm}},{\vec{y}}_{\text{sm}}$) plane.

Moreover, a metallic cross is fixed in front of the X-ray source. The axis passing both through the X-ray point source and the 3D intersection point of the metallic cross is perpendicular to the radiograph planes, to the $\left({\vec{x}}_{\text{sm}},{\vec{y}}_{\text{sm}}\right)$ plane of the first viewpoint and to the (${\vec{z}}_{\text{sm}},{\vec{y}}_{\text{sm}}$) plane of the second viewpoint. With this geometry, the projection of the axes ${\vec{x}}_{\text{sm}}$, ${\vec{y}}_{\text{sm}}$, and ${\vec{z}}_{\text{sm}}$ of the simulation machine coordinate system is visualized exactly by the projections of the metallic cross onto the radiographs.

The proposed positioning algorithm was tested in the simulation room.

### 3.2. Phantom description and CT-data

Tests were performed with a plaster head acting as phantom (see Figure 5). Fifteen metallic balls (simulating tumors) were included in the head. These radio-opaque balls, with a mean diameter of 5 mm, were regularly spaced and placed exactly on three orthogonal axes. Figure 6(a) gives the labels of these balls. It is noticeable that the balls are distributed into the whole head volume so that it can be checked if the positioning accuracy depends on the tumor localization.

A scan was performed with the plaster head placed in the CT-machine. The balls were spread out on several voxels of the CT. The mass centre positions (*X* ^*i*^_CT_, *Y* ^*i*^_CT_, *Z* ^*i*^_CT_) were computed for each ball *p*_*i*_(*i* ∈ [1, 15]) ).

### 3.3. First positioning test

The balls were successively placed at the simulation machine isocentre by superimposing the ball projections and the cross intersection projections viewed on the two control radiographs. This placement can be done very accurately by experienced radiotherapists. The laser positions on the plaster head were marked precisely for each ball placement on *O*_sm_. Thus, the placement of the marks on the laser beams ensures a very accurate positioning of the balls on the isocentre. If a ball is placed on *O*_sm_, then the positioning algorithm should ideally give (0,0,0) as result for the ball coordinates in $\left(O_{\text{sm}},{\vec{x}}_{\text{sm}},{\vec{y}}_{\text{sm}},{\vec{z}}_{\text{sm}}\right)$. It is noticeable that this positioning experiment is conducted like a true patient positioning in the therapy room.

The sensor was fixed in the simulation room and its position was calibrated in the $\left(O_{\text{sm}},{\vec{x}}_{\text{sm}},{\vec{y}}_{\text{sm}},{\vec{z}}_{\text{sm}}\right)$ coordinate system. The surface of the plaster head given by the CT-scan was registered with the plaster head's face acquired in the simulation room. The balls *p*_*i*_ were all placed at the isocentre and positions of their centres were computed with (9). The (*x* ^sm^_*i*,com_, *y* ^sm^_*i*,com_, *z* ^sm^_*i*,com_) ball coordinates in $\left(O_{\text{sm}},{\vec{x}}_{\text{sm}},{\vec{y}}_{\text{sm}},{\vec{z}}_{\text{sm}}\right)$ and their *d* ^sm^_*i*,com_ distances to *O*_sm_ are given in Table 2. The mean and standard deviation values of the *d* ^sm^_*i*,com_ distances are, respectively, 0.4 mm and 0.15 mm. With these results, several observations can be formulated.

The mean positioning error is very small and indicates a submillimetre accuracy.

No correlation can be established between tumor positions and positioning errors. In other terms, a weak variability affects the positioning accuracy when considering different head regions (head centre or skull region). This result is important since the lower this variability is, the more the positioning errors are predictable.

The voxel of the CT-modality having a size of 0.313 mm×0.313 mm ×2 mm means that the (*x* ^CT^_*i*,com_, *y* ^CT^_*i*,com_, *z* ^CT^_*i*,com_) centre coordinates of the balls (with a 5 mm diameter) are affected by errors. Theses errors have also an impact on the patient positioning accuracy. The positioning accuracy can still be improved with scanners (CT-modality or other modalities) delivering volume data with a higher resolution.

### 3.4. Second positioning test

The purpose of the second positioning test was the assessment of the variability of the positioning results with regards to the calibration data, the phantom data, and sensor viewpoint differences. Indeed, from one acquisition to another, the distributions of the 3D sensor points on the calibration piece spheres and on the plaster head are different, even if the point density remain quasiconstant. Concerning the viewpoint differences, acquisitions were performed for sensor/object distances ranging in [90,110] cm and for angle deviations (from reference angles) belonging in [ −10°, 10°].

Each ball was acquired several times for different angles of view. Images of the calibration piece were also taken for each sensor position. For each ball, the mean distance and the standard deviation were computed for the $d_{\text{sm}}^{i,\text{com}}$ distances to the isocentre. The standard deviation, acting as first criterion for the assessment of the isocentre/ball distance variability, was smaller than 0.1 mm for the fifteen balls. The values given in Table 3 for ball 3 are representative of the positioning algorithm variability. The standard deviation with respect to the mean value of the $d_{\text{sm}}^{3,\text{com}}$ distances of ball 3 is 0.055 mm. The mean distance between the mean position of a ball and the different positions of the same ball is another criterion allowing the assessment of the positioning variability. The mean distance between the positions of ball 3 and the (0.093, 0.024, 0.073) mean position coordinates of ball 3 is 0.18 mm. It is noticeable that the mean position of ball 3 is very close to the (0,0,0) isocentre coordinates. The small values obtained for the two algorithm variability criteria show that the positioning algorithm is relatively independent towards sensor position differences and different 3D point distributions. Moreover, it is noticeable that the sensor can be fixed once and for all in an optimal position in terms of patient positioning accuracy. Thus, the positioning accuracy dependency according to the sensor position is not a crucial problem.

### 3.5. Registration of human faces

The only step of the positioning algorithm which can lead to different results when human data is used instead of phantom data is the 3D surface registration. Two tests were carried out to assess the influence of the nonrigid cutaneous surface on the registration algorithm.

**First registration test**

**Figure 7:**
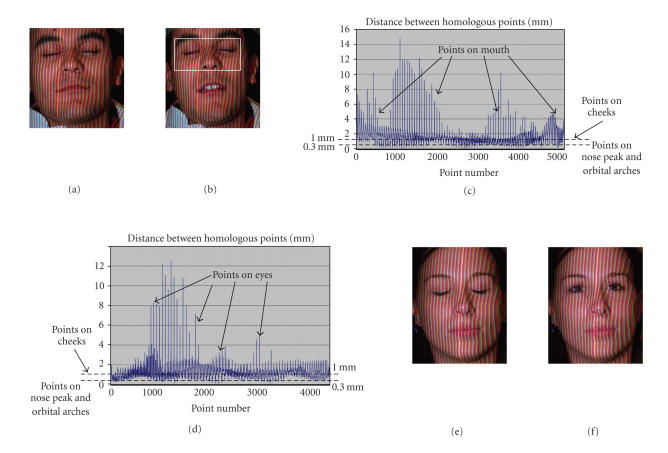
Registration results for humans. (a) Man with closed eyes and mouth. (b) Man with closed eyes and open mouth. The window in (b) indicates the data of the image to be transformed in the first registration test (the whole 3D points are used in the second test). The colour rays are the structured light information used for the 3D point reconstruction. (c) Computed distances between homologous points of (a) and (b) after registration. (d) Computed distances between homologous points of (e) and (f) after registration. (e) Woman with closed eyes and mouth. (f) Woman with open eyes and closed mouth.

A first image is acquired with the 3D sensor for a person who takes a neutral expression (eyes open in a natural way and closed mouth). This image simulates the CT-data. A second image was taken immediately after the first acquisition. Even if the person was asked to keep the same expression (the mouth remained closed), differences exist between the two images (eyelids more or less open, teeth more or less clenched, different point distribution over the face, etc.). In the second image, the data included in a window comprised between the bottom of the forehead and the bottom of the nose (see Figure 7(b)) was manually extracted (this face region can automatically be extracted by looking for the high curvature points corresponding to the nose and to the orbital arches). A transformation consisting of some decimetre translation components and of three rotation angles ranging each in [ −10°, 10°] is applied to the 3D surface extracted from the second image. The extracted data was then registered with the first image. This test was done for 15 women and men.

After registration, the distances between each point of the transformed surface (second image) and the corresponding computed points on the reference surface (closest points on the surface of the first image) are determined. The mean distance between these homologous points never exceeded 0.1 mm for all 15 people. It is noticeable that this mean value is only a little bit greater than the 0.03 mm inherent registration accuracy computed for the ideal phantom data (see Section 2.4.6). The 0.1 mm distances correspond to errors smaller than 1° and one tenth of millimetres for the angles and the translations, respectively. These results prove that the registration scheme based on the dHd is very robust and accurate, not only for phantom data, but also for human data. A high registration accuracy can be obtained since, in the considered window, the anatomical parts supporting the skin (orbital arches, nose and cheek-bone) are rigid surfaces. In this region, skin movements affect only slightly the 3D face shape. The mouth and the essential parts of the cheeks (nonrigid regions) are outside the window.

**Second registration test**

The aim of the second test is to assess the registration algorithm accuracy and robustness in more extreme situations. A first image is again acquired for people. For this reference image, the people systematically closed their eyes and their mouth (teeth slightly clenched). This face posture can easily be maintained. Other images were acquired for each person with different face configurations: closed mouth/open eyes, open mouth/closed eyes, and open mouth/open eyes. Transformations consisting of some decimetre translation components and of three rotation angles ranging each in [ −10°, 10°] are applied to these images. The latter are then registered with the reference image. The whole data of each image (no data extraction) was used during the registration.

Figure 7 illustrates typical results obtained with different men and women. Figure 7(c) allows a quantitative assessment of the registration quality of the 3D data represented by the images of Figures Figure 7(a) and 7(b). The graphic of Figure 7(c) gives, for each point of the transformed image, the shortest distance to the surface of the model image. The distances between these homologous points vary greatly according to the face region. It was verified that the distances between homologous points located around the mouth or on the chin, on the cheeks, and on the regions close to the nose peak or orbital arches are, respectively, greater than 3 mm, range approximatively in [0.3, 3] mm or are smaller than about 0.3 mm. These observations are coherent since

- if the images are well registered, big differences exist for the mouth and the chin due to unconscious movements,
- some millimetre variability is normal for points located on cheeks which have a low rigidity, and
- small errors for points located on the nose peak and on the orbital arches are predictable since these face parts are the most rigid (opening the mouth does not normally change the nose position).

If only the points on the nose peak and around the orbital arches are considered, the mean ${\bar{d}}_{\text{rigid}}$ distance between homologous points after registration is 0.09 mm. The ${\bar{d}}_{\text{all}}$ mean distance computed for all points is 1.6 mm. Meanwhile, the last measure is strongly influenced by the points located around the mouth and on the chin. Without these last points, the ${\bar{d}}_{\text{cheeks}}$ mean distance including the cheek points is 1.05 mm. However, the ${\bar{d}}_{\text{rigid}}$ measure is the most pertinent (since it is based on rigid face parts) and indicates that the registration had a submillimetre accuracy for the man of Figure 7. It is noticeable that the ${\bar{d}}_{\text{rigid}}$ value is close to the 0.1 mm mean distance obtained for the first registration test with the window.

The same observation can be made for the woman of Figures Figure 7(e) and Figure 7(f) with closed eyes (reference image) and open eyes (image to be transformed). After the registration the ${\bar{d}}_{\text{all}}$, ${\bar{d}}_{\text{cheeks}}$, and ${\bar{d}}_{\text{rigid}}$ mean distances are 1.2 mm, 0.99 mm and 0.11 mm, respectively. ${\bar{d}}_{\text{rigid}}$ indicates again a submillimetre registration accuracy. Similar results were obtained for all people, even if both the eyes and mouth were open.

**Registration result discussion**

The first tests presented here indicate that the 3D points between the bottom of the forehead and of the nose should systematically be extracted from the data set to be transformed before registering it with the model data (whole face points). It is recalled that this face part is always visible when the devices defined in section 1.3 are used. With this way to proceed, the distances between the homologous face points have a very small mean value (0.1 mm). The tests also proved that eyelid movements have a negligible impact on the registration accuracy. The tests with the phantom demonstrated that there is no correlation between the positioning accuracy and the lesion position in the head (Section 3.4). This fact indicates that if the lesion is close to the face surface (0.1 mm error) or in the head centre (two very different localizations), the lesion localization error due to the registration is always about 0.1 mm (or at least by far smaller than 1 mm). Thus, the first advantage of the dHd taken as similarity measure lies in the fact that small face surfaces lead to an accurate registration. The only condition is that face regions with enough geometrical information are included (regions with high curvatures like the nose or orbital arches) in the data. Another advantage of the dHd is its ability to register two surfaces of different sizes and point densities (this measure is often used when surface data of two different modalities must be registered).

The second tests proved that the dHd is able to register two surfaces presenting large geometrical differences while ensuring submillimetre alignment accuracy. In fact, the tests confirmed that the dHd can handle data containing outliers (points on the mouth or on the eyes in the case of very strong eyelid movement) without greatly affecting the registration accuracy.

The tests also proved that the registration algorithm converges in a robust way towards the solution, even for big head position differences between the two modalities. Moreover, neither an initial manual alignment nor an initial homologous point marking is required. According to the literature, the dHd leads to registration accuracies which are almost independent of the translation differences between surfaces. This fact was confirmed by the results. Orientation differences (around each axis) ranging in [ −10°, 10°] were always successfully treated by the registration algorithm. The tested position differences are greater than those encountered in clinical situations. In fact, radiotherapists place the patient with initial errors of one centimetre (or a few millimetres) and some degrees in terms of translations and rotations. The proposed registration scheme is able to handle bigger differences in an automatic way.

## 4. CONCLUSION

The results presented in this contribution prove that the proposed algorithm is an important first step towards a patient positioning which allows for the association of CRT and FRT in the case of intracranial lesion irradiation. Tests with a phantom proved that the inherent accuracy of the whole positioning algorithm (sensor calibration and registration) is 0.4 mm. Registration tests with human data proved that the mean alignment errors are very small (about one tenth of millimetres). This registration accuracy leads us to think that the whole positioning method will also lead to a submillimetre accuracy for patient data. In fact, as suggested by Li et al. [12], if the calibration and the registration have each a submillimetre accuracy, the limitation in terms of precision is rather due to the precision of the patient immobilization devices than to positioning algorithm precision. The fact that Li obtained a submillimetre accuracy with similar algorithm principles and sensors indicates that it is also possible to reach a submillimetre accuracy for patients. The next step of the positioning algorithm evaluation will consist in experiments conducted as follows. Patients will be positioned with the classical invasive frame-based method. The proposed algorithm will be used in parallel to obtain a second tumor coordinate set. The later coordinates will be compared to those given by the frame based method. Control radiographs will also be used to test the positioning accuracy of the algorithm with patient data.

The proposed method is noninvasive and no dedicated piece must be built for patients. Standard treatment protocols are not influenced by the algorithm. Moreover, only conventional and simple immobilization devices are required. The drawbacks relating to frames or face masks are avoided.

One of the main results of this contribution lies in the performances of the registration algorithm. The optimization method converges robustly and accurately towards the solution, even for large head position differences. Facial expression changes can also be processed by the algorithm.

Phantom-based tests proved that the positioning accuracy does not depend on the lesion position in the head. The fact that the irradiation must be done with well-known errors (at least submillimetre errors) explains why it is important for the positioning accuracy to be independent of the lesion localization.

## Figures and Tables

**Table 1:** Inherent accuracy of the registration algorithm according to the data down-sampling parameter *ε*.

${\tilde{T}}_{\text{test}}^{-1}$ parameter values	${\tilde{T}}_{\text{test}}^{-1}-$ ${\tilde{T}}_{\text{CT},3\text{DS}}$ (*ε* = 0)	${\tilde{T}}_{\text{test}}^{-1}-$ ${\tilde{T}}_{\text{CT},3\text{DS}}$ (*ε* = 0.01)	${\tilde{T}}_{\text{test}}^{-1}-$ ${\tilde{T}}_{\text{CT},3\text{DS}}$ (*ε* = 0.1)	${\tilde{T}}_{\text{test}}^{-1}-$ ${\tilde{T}}_{\text{CT},3\text{DS}}$ (*ε* = 0.5)
*θ* = 7°	0.9	0.91	0.91	0.92
*ϕ* = 9°	−0.6	−0.6	−0.62	−0.62
*ψ* = −5°	1.09	1.09	1.58	1.06
*t* ^reg^_*x*_ = 50 mm	−0.02	−0.02	−0.03	−0.03
*t* ^reg^_*y*_ = −40 mm	−0.01	−0.01	− 0.01	−0.01
*t* ^reg^_*z*_ = 50 mm	0.06	0.06	0.06	0.06

**Table 2:** First positioning test results. The *i* -ball labels are those of Figure 6 (all values are given in millimeters).

*i*	*x* ^*i*,com^_sm_	*y* ^*i*,com^_sm_	*z* ^*i*,com^_sm_	*d* ^*i*,com^_sm_
1	0.13	−0.08	0.12	0.19
2	0.11	0.12	0.18	0.24
3	0.18	−0.02	0.04	0.19
4	0.47	0.11	0.35	0.59
5	−0.27	−0.10	−0.07	0.31
6	0.29	0.02	−0.18	0.34
7	0.61	0.29	0.31	0.74
8	−0.21	0.17	0.15	0.31
9	0.31	0.06	−0.18	0.36
10	0.18	−0,31	0.32	0.48
11	0.26	−0.34	0.08	0.44
12	0.31	0,21	0.28	0.47
13	−0.16	0.21	0.42	0.51
14	0.15	0.23	−0.19	0.33
15	0.36	−0.17	0.28	0.49

**Table 3:** Ball 3 positioning results for different acquisitions and 3D sensor viewpoints. *d* ^3,com^_sm_ is the distance between the ball with coordinates (*x* ^3,com^_sm_, *y* ^3,com^_sm_, *z* ^3,com^_sm_) and the isocentre (All values are given in millimeters).

Acquisition number	*x* ^3,com^_sm_	*y* ^3,com^_sm_	*z* ^3,com^_sm_	*d* ^3,com^_sm_
1	0.24	0.12	0.01	0.27
2	0.11	0.01	0.03	0.11
3	0.11	0.12	0.21	0.27
4	0.18	−0.02	0.04	0.19
5	0.14	0.13	0.22	0.29
6	−0.21	−0.07	0.12	0.25
7	0.03	0.15	−0.08	0.24
8	0.17	−0.08	0.11	0.22
9	0.08	−0.14	0.10	0.19

## Footnotes

3D flash! cam system from 3D metrics, Petaluma, CA 94954,USA.

## References

[1] Gross MW, Spahn U, Engenhart-Cabillic R (2003). Assessment of the accuracy of a conventional simulation for radiotherapy of head and skull base tumors. *Technology in Cancer Research and Treatment*.

[2] Gill SS, Thomas DGT, Warrington AP, Brada M (1991). Relocatable frame for stereotactic external beam radiotherapy. *International Journal of Radiation Oncology, Biology, Physics*.

[3] Hariz MI, Henriksson R, Löfroth P-O, Laitinen LV, Säterborg NE (1990). A non-invasive method for fractioned stereotactic irradiation of brain tumors with linear accelerators. *Radiotherapy and Oncology*.

[4] Delannes M, Daly N, Bonnet J, Sabatier J, Tremoulet M (1991). Fractioned radiotherapy of small inoperable lesions of the brain using a non-invasive stereotactic frame. *International Journal of Radiation Oncology, Biology, Physics*.

[5] Graham JD, Warrington AP, Gill S, Brada M (1991). A non-invasive, relocatable stereotactic frame for fractionated radiotherapy and multiple imaging. *Radiotherapy and Oncology*.

[6] Uematsu M, Shioda A, Suda A (2001). Computed tomography-guided frameless stereotactic radiotherapy for stage I non-small cell lung cancer: a 5-year experience. *International Journal of Radiation Oncology, Biology, Physics*.

[7] Mavroidis C, Flanz J, Dubowsky S, Drouet P, Goitein M (1998). High performance medical robot requirements and accuracy analysis. *Robotics and Computer-Integrated Manufacturing*.

[8] Van Lin ENJTh, van der Vight L, Huizenga H, Kaanders JHAM, Visser AG (2003). Set-up improvement in head and neck radiotherapy using a 3D off-line EPID-based correction protocol and a customised head and neck support. *Radiotherapy and Oncology*.

[9] Sarrut D, Clippe S (2000). Patient positioning in radiotherapy by registration of 2D portal to 3D CT images by a contend-based research with similarity measures.

[10] Bansal R, Staib LH, Chen Z Entropy-based, multiple-portal-to-3D CT registration for prostate radiotherapy using iteratively estimated segmentation.

[11] Meeks SL, Bova FJ, Wagner TH, Buatti JM, Friedman WA, Foote KD (2000). Image localization for frameless stereotactic radiotherapy. *International Journal of Radiation Oncology, Biology, Physics*.

[12] Li S, Liu D, Yin G, Zhuang P, Geng J (2006). Real-time 3D-surface-guided head refixation useful for fractionated stereotactic radiotherapy. *Medical Physics*.

[13] Djajaputra D, Li S (2005). Real-time 3D surface-image-guided beam setup in radiotherapy of breast cancer. *Medical Physics*.

[14] Nelder JA, Mead R (1965). A simplex method for function minimization. *Computer*.

[15] Mäkelä T, Clarysse P, Sipilä O (2002). A review of cardiac image registration methods. *IEEE Transactions on Medical Imaging*.

[16] van den Elsen PA, Pol EJD, Viergever MA (1993). Medical image matching-a review with classification. *IEEE Engineering in Medicine and Biology Magazine*.

[17] Maintz JBA, Viergever MA (1998). A survey of medical image registration. *Medical Image Analysis*.

[18] Efrat A, Itai A Improvements on bottleneck matching and related problems using geometry.

[19] Kirchberg KJ, Jesorsky O, Frischholz R Genetic model optimization for Hausdorff distance-based face localization.

[20] Indyk P, Venkatasubramanian S Approximate congruence in nearly linear time.

[21] Pollard AE, Barr RC (1987). Adaptive sampling of intracellular and extracellular cardiac potentials with the fan method. *Medical and Biological Engineering and Computing*.

